# Prevalence of musculoskeletal disorders and rheumatic disease in the Warao, Kari’ña, and Chaima indigenous populations of Monagas State, Venezuela

**DOI:** 10.1007/s10067-016-3194-0

**Published:** 2016-02-19

**Authors:** Ysabel Granados, Celenia Rosillo, Ligia Cedeño, Yanira Martínez, Gloris Sánchez, Geovalis López, Fernando Pérez, Damarys Martínez, Gabriela Maestre, Sol Berbin, Rosa Chacón, Iván Stekman, Evart Valls, Ingris Peláez-Ballestas

**Affiliations:** Rheumatology Unit, Hospital Dr. Manuel Núñez Tovar, Maturín, Monagas Venezuela; Centro Medico, 5º Piso Consultorio 4, Sector Las Avenidas, Maturín, Estado Monagas 6201 Venezuela; Health Network “Barrio Adentro”, Ministerio del Poder Popular para la Salud, Estado Monagas, Venezuela; Rheumatology Unit, Hospital Central de Maracay, Maracay, Venezuela; IVSS “Dr. Cesar Rodríguez”, Puerto La Cruz, Venezuela; Hospital “Dr. José A. Urrestarazu”, Caripe, Estado Monagas Venezuela; Private Clinical Center, Caracas, Venezuela; Hospital Universitario de Caracas, Caracas, Venezuela; Health Indigenous Department, Estado Monagas, Venezuela; Rheumatology Unit, Hospital General de Mexico “Dr. Eduardo Liceaga”, Mexico City, Mexico

**Keywords:** Indigenous populations, Musculoskeletal diseases, Prevalence, Rheumatic diseases, Venezuela

## Abstract

This study aimed to estimate the prevalence of musculoskeletal disorders and rheumatic diseases in the Warao, Kari’ña, and Chaima indigenous populations of Monagas State, Venezuela. A cross-sectional, analytical, community-based study was conducted in 1537 indigenous subjects ≥18 years old (38.6 % male, mean age 41.4 ± 17.5 years). The cross-culturally validated Community Oriented Program for the Control of Rheumatic Diseases (COPCORD) diagnostic questionnaire was applied. Subjects with a positive COPCORD diagnosis (either historic or current pain) were evaluated by primary care physicians and rheumatologists. A descriptive analysis was performed and comparisons made using analysis of variance and the chi-square test. Pain in the last 7 days was reported by 32.9 %, with pain intensity, according to a Likert-type scale [no pain, 195 (38.5 %); minimal pain, 231 (45.6 %); strong pain, 68 (13.4 %); intense pain, 5 (0.9 %)], 38.0 % reported historical pain, and 641 (41.7 %) had either historic or current pain. Of the COPCORD-positive subjects, pain most frequently occurred in the knee, back, and hands. Musculoskeletal and rheumatic diseases included osteoarthritis (14.1 %), back pain (12.4 %), rheumatic regional pain syndromes (RRPS) (9.7 %), undifferentiated arthritis (1.5 %), rheumatoid arthritis (1.1 %), and fibromyalgia (0.5 %). Chaima (18.3 %) and Kari’ña (15.6 %) subjects had a high prevalence of osteoarthritis, and Warao subjects had a high prevalence of low back pain (13.8 %). The prevalence of RRPS was high in all three ethnic groups. The Chaima group had the highest prevalence of rheumatic diseases, with 2.0 % having rheumatoid arthritis. This study provides useful information for health care policy-making in indigenous communities.

## Introduction

The prevalence and severity of rheumatic diseases and musculoskeletal (MSK) disorders may vary according to ethnicity and socioeconomic conditions [1, 2]. Genetic and epidemiological studies have indicated that certain rheumatic conditions may have greater prevalence and severity in specific indigenous groups [3]. Indigenous populations are numerically small and have diverse genetic and cultural characteristics [4]. The expansion of Westernized societies to their communities and historically established territories [5] constitutes a serious threat to their cultural heritage and lifestyles, which may have a negative influence on the health of these communities. The impact of chronic diseases on overall health is still unknown in many countries with indigenous populations, such as in Latin American countries.

Venezuela has 30 million inhabitants [6], with 724,592 indigenous subjects divided into 34 ethnic groups in 10 of the 23 states in the country. The State of Monagas located in eastern Venezuela has 17,898 indigenous (3.6 % of the state’s total population) grouped into three ethnicities (Warao, Kari’ña, and Chaima) [7]. The main causes of mortality in these groups are known [8], but there is little epidemiological information about the impact of MSK disorders and rheumatic diseases on these populations, although these are among the most prevalent chronic and disabling conditions in medical practice [9].

The Community Oriented Program for the Control of Rheumatic Diseases (COPCORD) questionnaire has been developed as a means of estimating the prevalence of rheumatic diseases [10] and MSK pain in various regions of the world [11, 12]. This methodology arose from an initiative by the International League of Associations for Rheumatology and the World Health Organization (WHO) to increase research on these chronic diseases in rural, urban, or marginal minority communities [13].

Epidemiological studies in indigenous populations need to overcome limitations such as community distrust, limited access to primary health care, and barriers to participation arising from beliefs, cultures, and languages [14, 15]. These limitations, however, should not deter the conduction of health studies, which can provide evidence for the development of culturally sensitive health policies. Thus, the objective of this study was to use the COPCORD methodology in order to estimate the prevalence of MSK disorders and rheumatic diseases in the Warao, Kari’ña, and Chaima indigenous people of Monagas State, Venezuela.

## Material and methods

### Design and population

A cross-sectional, descriptive, community-based study was performed in natives ≥18 years of age in the Warao, Kari’ña, and Chaima indigenous groups in Monagas State, Venezuela. Subjects were defined as indigenous if they were descended from indigenous people, lived in the indicated geographical area, and maintained the cultural, social, and economic identity of his/her people or community, even when adopting elements from other cultures [16]. The sample was based on a community census

The Warao are the largest ethnic group in Monagas and the second largest in Venezuela [17]. They have inhabited the Orinoco River delta for over 8000 years and maintain their language and many of their ancient customs. They form a peaceful society of fishermen and harvesters settled in communities that are mostly only reached by boat. The Kari’ña inhabit the eastern plains and jungle regions of Bolivar district [18]. In Monagas, there is a population of 1174 who retain their native customs and language. Agriculture is their main livelihood, and they are adept craftsmen, making baskets and hammocks. The Chaima live in inaccessible mountainous areas [19] in the Turumiquire Mountains of the Andean coastal system [20], where they farm and produce handicrafts. The Chaima identity had become obscured and their language was in serious danger of extinction, but in the last two decades, a recovery and ethnogenesis process has been initiated.

### Instrument

The cross-culturally validated COPCORD questionnaire adapted for Venezuela [21, 22] was applied. The questionnaires were administered in a door-to-door survey by primary care physicians, accompanied by previously trained indigenous male nurses from the respective ethnic groups, who were bilingual. All natives ≥18 years old who had lived in their community for more than 6 months at the time of the study were surveyed.

### Identification of cases with musculoskeletal disorders

The questionnaire included questions related to symptoms (pain, stiffness, and disability), treatment, adaptation to the problem, medical help sought, and non-conventional medicine use. The COPCORD questionnaire was considered positive when the participant subject reported MSK pain during the last 7 days and/or a history of pain.

### Clinical evaluation

All COPCORD-positive individuals were evaluated in duplicate, by primary care physicians and certified rheumatologists. The American College of Rheumatology criteria were used for the diagnosis of osteoarthritis of the hands and knees [23, 24], rheumatoid arthritis [25], fibromyalgia [26], and systemic lupus erythematosus [27]. The Wallace criteria for diagnosis of gout [28], the modified New York criteria for ankylosing spondylitis [29], and the Southampton Group criteria for rheumatic regional pain syndromes (RRPS) [30] were used. Any non-specific joint or muscle pain, or those not meeting the classification criteria, were designated as MSK disorders and classified according to the WHO International Classification of Diseases version 10 [31]. The administration of the instrument and the evaluation of the COPCORD-positive patients were performed on the same day. If the patient required laboratory tests or a radiological study, he/she was referred to the Rheumatology Unit of Hospital “Dr. Manuel Núñez Tovar” in Maturín, Monagas State.

### Ethics

The study was approved by the Ethics and Research Committee of the University Hospital “Dr. Manuel Núñez Tovar” and by representatives of the indigenous communities, thus complying with the provisions of the Communities and Indigenous Peoples Organic Law of the Bolivarian Republic of Venezuela [16]. All participants were informed about the study and gave their consent for use of their anonymized data.

### Statistical analysis

Microsoft Access for Windows was used for collection of the encoded information, followed by data cleanup, and an exploratory analysis of the variables. This reported the distribution of continuous variables, and absolute and relative ordinal, nominal, or categorical frequencies. A bivariate analysis was performed on each of the study variables. One-way or two-way analysis of variance was used for comparisons of continuous variables and the chi-square test for ordinal, nominal, or categorical variables. The statistical analysis package STATA 11 (Stata Corp., College Station, TX, USA) was used.

## Results

A total of 1537 subjects (100 % response rate) took part in the survey, including 943 (61.4 %) women and 594 (38.6 %) men with a mean age (±standard deviation) of 41.4 ± 17.5 years. The mean duration of education was 4.5 ± 4.4 years; 21.1 % were peasants, 17.7 % were housewives, 16.6 % were artisans, and 1.5 % were teachers. Only 0.3 % were professionals (Table 1). The Chaima group was mainly engaged in farming, often transporting heavy goods on their heads, shoulders, or backs through mountainous terrain. The Kari’ña were characterized by their handicraft work (fabrics), as were the Warao; thus, they undertook more repetitive movements and less physical activity.

**Table 1:** Population’s social-demographic data (*n* = 1537)

Demographic characteristics	(*n* = 1537)
Gender, *n* (%)	
Female	943 (61.4)
Male	594 (38.6)
Age, average (DE; range)	41.4 (17.5; 18–96)
Schooling, average (DE; range)	4.5 (4.4; 0–16)
Marital status, *n* (%)	
Single	262 (17.0)
Married/free union	1063 (69.1)
Widower	30 (1.9)
Separated	182 (11.8)
Ethnic group, *n* (%)	
*Chaima*	692 (45.0)
*Kari’ña*	262 (17.0)
*Warao*	583 (37.9)
Language, *n* (%)	
Spanish	1429 (92.7)
Native language	621 (40.4)
Kinship, *n* (%)	
Indigenous father	1306 (84.9)
Indigenous mother	1437 (93.4)
Work (yes), *n* (%)	951 (61.8)
Occupation, *n* (%) (*n* = 1400)	
Peasant	296 (21.1)
Housewife	248 (17.7)
Artisan	233 (16.6)
None	202 (14.4)
Non-specified workers	100 (7.1)
Laborer	81 (5.7)
Fisherman	74 (5.2)
Businessman	55 (3.9)
Domestic service	44 (3.1)
Others^a^	24 (1.7)
Teacher	21 (1.5)
Technicians	17 (1.2)
Professionals	5 (0.3)
Physical activity	
Load over 4 kg (8.82 lb)	486 (31.6)
Repeatability	345 (22.4)

^a^Seamstress, administrative workers; DE (abbreviation)

### Pain characteristics

Pain in the last 7 days was reported by 505 (32.9 %) subjects; 584 subjects (38.0 %) had suffered pain at some time in their life (historic), and 641 (41.7 %) had either historic or current pain (COPCORD-positive). Pain intensity in the last 7 days was reported in a Likert-type scale, as follows: no pain, 195 (38.5 %); minimal pain, 231 (45.6 %); strong pain, 68 (13.4 %); and intense pain, 5 (0.9 %), with a median score of 0 and interquartile range of 0 to 0.2 (Table 2). The treatments administered were as follow: non-steroidal anti-inflammatory drugs 276/382 (72.2 %), analgesics 61/382 (15.9 %), and 7/382 (1.5 %) received disease-modifying antirheumatic drugs.

**Table 2:** Social-demographic characteristics and musculoskeletal pain prevalence by indigenous group

Variables	Total *n* = 1537	Warao *n* = 583 *n* (%)	Chaima *n* = 692 *n* (%)	Kari’ña *n* = 262 *n* (%)	*p*
Social-demographics					
Age, average (SD)^a^	41.4 (17.5)	37.9 (15.6)	44.2 (18.2)	41.5 (18.4)	<0.001
Gender (female), *n* (%)	943 (61.3)	352 (60.3)	416 (60.1)	175 (66.7)	0.130
Schooling average (SD)^a^	4.5 (4.4)	2.1 (3.0)	5.5 (4.3)	7.3 (4.9)	< 0.001
Language, *n* (%)					
Spanish	1429 (92.7)	479 (82.1)	691 (99.8)	259 (98.8)	<0.001
Indigenous language	621 (40.4)	567 (97.2)	4 (0.5)	50 (19.0)	<0.001
Kinship n (%)					
Indigenous father	1306 (84.9)	537 (92.1)	571 (82.5)	198 (75.5)	<0.001
Indigenous mother	1437 (93.4)	574 (98.4)	645 (93.2)	218 (83.2)	<0.001
Physical activity, *n* (%)					
Load over 4 kg (8.82 lb)	345 (22.4)	283 (48.5)	13 (1.8)	49 (18.7)	<0.001
Repeatability	486 (31.6)	149 (25.5)	268 (38.7)	69 (26.3)	<0.001
MSK pain, *n* (%)					
MSK pain 7 days, *n* (%)	505 (32.8)	146 (25.0)	289 (41.7)	70 (26.7)	<0.001
Intensity, *n* (%)					
No pain	195 (38.5)	23 (15.7)	172 (59.5)	–	<0.001
Minimal pain	231 (45.6)	76 (52.0)	115 (39.7)	40 (57.1)	<0.001
Strong pain	68 (13.4)	41 (28.0)	1 (0.3)	26 (37.1)	<0.001
Intense pain	5 (0.9)	1 (0.6)	–	4 (5.7)	–
Historic MSK pain, *n* (%)	584 (38.0)	160 (27.4)	319 (46.1)	105 (40.0)	< 0.001
Intensity, *n* (%)b	*n* = 583*	*n* = 159*			
No pain	149 (25.5)	21 (13.2)	128 (40.1)	–	<0.001
Minimal pain	358 (61.4)	98 (61.6)	191 (59.8)	69 (65.7)	–
Strong pain	4 (0.6)	1 (0.6)	–	3 (2.8)	–
Intense pain	72 (12.3)	39 (24.5)	–	33 (32.3)	<0.001
Functional capacity (HAQ) median (RIQ)^b^	0 (0–0.2)	0 (0–0.2)	0.2 (00.6)	0 (0–0)	<0.001
Functional limitation, *n* (%)					
With no limitation	431 (68.0)	98 (68.0)	200 (70.4)	44 (62.8)	
Limitation in the past	148 (23.3)	27 (18.7)	73 (17.1)	12 (17.1)	
Current limitation	54 (8.5)	19 (13.1)	11 (3.8)	14 (20.0)	<0.001
Help care seeking for MSK complaint, *n* (%)	*n* = 525				
No attention	150 (28.5)	112 (58.9)	4 (1.7)	34 (31.4)	
Medical	250 (47.6)	–	222 (97.8)	28 (25.9)	
Health center	30 (5.7)	30 (15.7)	–	–	
Self-medication	1 (0.1)	–	1 (0.4)	–	
Traditional medicine	13 (2.4)	11 (5.7)	–	2 (1.8)	
Others	81 (15.4)	37 (19.4)		44 (40.7)	<0.001
Treatment for MSK complaint, *n* (%)	354 (23.0)	63 (32.8)	225 (66.9)	63 (55.7)	<0.001
Coping MSK complaint, *n* (%)		*n* = 192	*n* = 337	*n* = 113	
Not adapted	109 (16.9)	34 (17.7)	48 (14.2)	27 (23.8)	<0.001
Adapted	533 (83.0)	158 (82.2)	289 (85.7)	86 (76.1)	–

*MSK* musculoskeletal, *HAQ* Health Assessment Questionnaire, *IQR* interquartile range (25–75 %), *SD* standard deviation

^a^ANOVA Bonferroni test

^b^Kruskal–Wallis test

**Fig. 1:**
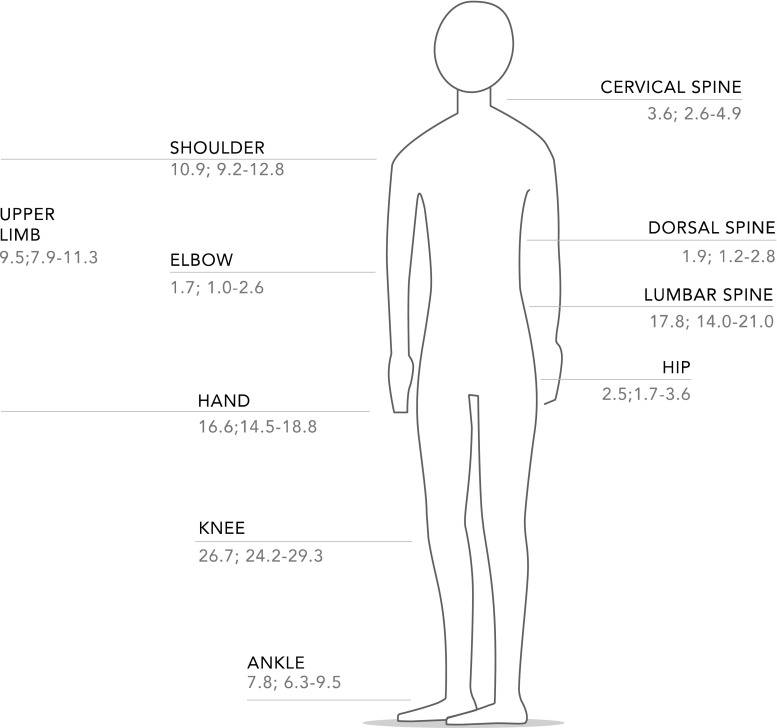
Musculoskeletal pain places in the last 7 days

**Fig. 2:**
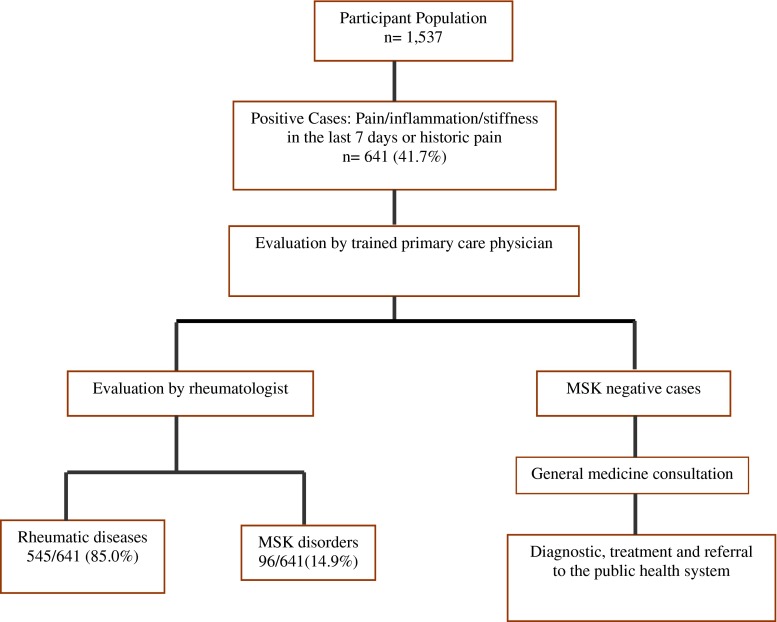
Rheumatic diseases detection

The most common sites of MSK pain were the knee, lumbar spine, hands, and ankles (Fig. 1). Of the 641 COPCORD-positive cases, a diagnosis of rheumatic disease was established in 545 (85 %; Fig. 2).

### Prevalence of rheumatic diseases

The prevalence of specific inflammatory rheumatic diseases in the three communities was as follows: osteoarthritis, 14.1 %; back pain, 12.4 %; RRPS, 9.7 %; undifferentiated arthritis, 1.5 %; rheumatoid arthritis, 1.1 %; and fibromyalgia, 0.5 %. The differences in prevalence between the communities are presented in Table 3.

**Table 3:** Rheumatic diseases prevalence in the Warao, Chaima, and Kari’ña indigenous groups

Rheumatic diseases	Total (1537) (%; IC 95 %)	Warao *n* = 583 (%; IC 95 %)	Chaima *n* = 692) (%; IC 95 %)	Kari’ña *n* = 262 (%; IC 95 %)	*p*
Osteoarthritis	217 (14.1; 12.4–15.9)	49 (8.4; 6.2–10.9)	127 (18.3; 15.5–21.4)	41 (15.6; 11.4–20.6)	<0.001
Back pain	192 (12.4; 10.8–14.2)	81 (13.8; 11.1–16.9)	86 (12.4; 10.0–15.1)	25 (9.5; 6.2–13.7)	0.2
RRPS ^a^	150 (9.7; 8.3–11.3)	44 (7.5; 5.5–9.9)	74 (10.6; 8.4–13.2)	32 (12.2; 8.5–16.8)	0.05
Undifferentiated arthritis	24 (1.5; 1.0–2.3)	5 (0.8; 0.2–9.9)	16 (2.3; 1.3–3.7)	3 (1.1; 0.2–3.3)	0.09
Rheumatoid arthritis ^b^	17 (1.1; 0.6–1.7)	3 (0.5; 0.1–1.4)	14 (2.0; 1.1–3.3)	–	<0.001
Fibromyalgia	9 (0.5; 0.2–1.1)	–	8 (1.1; 0.5–2.2)	1 (0.3; 0.0009–2.1)	0.02
Spondylo-Arthritis ^c^	7 (0.4; 0.1–0.9)	–	5 (0.7; 0.2–1.6)	2 (0.7; 0.09–2.7)	0.1
Gout	5 (0.3; 0.1–0.7)	1 (0.1; 0.004–0.9)	3 (0.4; 0.08–1.2)	1 (0.3; 0.009–2.1)	0.1
Systemic lupus erythematosus	1 (0.06; 0.001–0.3)	–	1 (0.1; 0.003–0.8)	–	–
Scleroderma	1 (0.06; 0.001–0.3)	–	–	1 (0.3,0.009–2.1)	–
MSK ailments	100 (6.5; 5.3–7.8)	23 (3.9; 2.5–5.8)	53 (7.6; 5.7–9.8)	24 (9.1; 5.9–13.3)	0.004

^a^Rheumatic regional pain syndromes

^b^A Juvenile idiopathic arthritis was included

^c^Two ankylosing spondylitis, four psoriatic arthritis, and one undifferentiated spondyloarthritis

Mean age and sex ratio varied according to type of rheumatic disease. Osteoarthritis was more common in women (62.6 %), and mean age was 62.4 years, while lumbar pain was more common in men (52.6 %), and mean age was 40.5 years old. Most patients with rheumatoid arthritis adapted to pain (87.5 %) and disability (26.6 %), as shown in Table 4.

**Table 4:** Social-demographic characteristics and most prevalent rheumatic diseases

Variables	Osteoarthritis *n* = 217	Back pain *n* = 192	Rheumatoid arthritis *n* = 17	Undifferentiated arthritis *n* = 24
Age, average (SD)	62.4 (14.2)	40.5 (14.4)	54.3 (16.5)	37.5 (11.6)
Gender (female), *n* (%)	136 (62.6)	91 (47.4)	14 (87.5)	20 (83.3)
Schooling median (IQR)	2 (0–4)	4 (0–6)	1.5 (0–5)	5 (0–8)
Indigenous language, *n* (%)	59 (27.1)	80 (41.6)	3 (18.5)	6 (25.0)
Load over 4 kg	83 (38.2)	76 (39.5)	2 (12.5)	7 (29.1)
Repeatability	37 (17.0)	41 (21.3)	3 (18.5)	6 (25.0)
Help care seeking for MSK complaint, *n* (%)	*n* = 187	*n* = 153		
No attention	30 (20.3)	58 (37.9)	–	5 (26.3)
Medical	101 (55.4)	54 (35.2)	12 (80.0)	12 (63.1)
Health Center	9 (4.9)	15 (9.8)	3 (14.0)	1 (5.2)
Self-medication	–	1 (0.6)	–	–
Traditional medicine	6 (3.3)	3 (1.9)	–	–
Others	29 (15.9)	22 (14.3)	–	1 (5.2)
Coping MSK complaint, *n* (%)				
Not adapted	31 (14.2)	36 (18.7)	2 (12.5)	21 (87.5)
Adapted	186 (85.7)	156 (81.2)	14 (87.5)	3 (12.5)
Functional limitation, *n* (%)	*n* = 216	*n* = 190		
With no limitation	140 (64.8)	138 (72.6)	2 (13.3)	19 (79.1)
Limitation in the past	56 (25.9)	39 (20.5)	9 (60.0)	2 (8.3)
Current limitation	20 (9.2)	13 (6.8)	4 (26.6)	3 (12.5)

*IQR* interquartile range (25–75 %), *SD* standard deviation

## Discussion

In this study, the prevalence of MSK pain in the last 7 days was 32.9 %, which was higher than that previously reported (22.4 %) in the Mestizo urban population of Monagas State, Venezuela [32], and similar to that reported in Australian aboriginals (33 %) [33]. The differences in prevalence reported by the three Venezuelan indigenous groups were likely related to different characteristics in their habits, habitat, attitude to health, and ways of dealing with illness and pain.

The Chaima had a higher prevalence of recent (41.7 %) and historical (46.1 %) pain, but lower current functional limitation (3.8 %), compared with the Warao and Kari’ña groups. Customs and language barriers appeared to affect pain perception among the groups, while the Warao and Kari’ña groups reported greater pain intensity, the Chaima, perhaps because of their more Westernized customs, showed greater concern in solving their health problems, with 97.8 % seeking medical care and treatment for their ailments. There was a significant difference between the three indigenous groups in their reported physical activity, which was related to the work performed by each ethnic type and their communities’ geographic location.

The knees were the most common articular region of pain, followed by the lumbar spine. These findings were similar to those reported in a non-indigenous population of Monagas State [32], in urban populations in Latin American countries such as Mexico [34] and Peru [35], and in the urban population of Shanghai, China [36].

The Chaima group had a higher prevalence of rheumatic diseases; osteoarthritis was present in 18.3 %, similar to that reported in aboriginal Australians (18 %) [32]. The prevalence of back pain and RRPS was higher in all three groups than reported in Australian aborigines [32]. The prevalence of rheumatoid arthritis in the Chaima group (2.0 %) was higher than that in the Warao (0.5 %) and Kari’ña (0 %) groups, higher than that reported in the Kaqchiquel ethnic group in Guatemala (0.53 %) [37], similar to that recorded in a non-indigenous group in Tucumán, Argentina (1.97 %) [38], but lower than that estimated in an urban population of Yucatan, Mexico (2.8 %) [39]. Other rheumatic diseases such as systemic lupus erythematosus had only been reported in the Chaima group (0.1 %), while none had been reported in Australian aborigines, the Kaqchiquel ethnic group in Guatemala, and Latin American indigenous groups [2]. Chaima territory had some colonization by Europeans predominantly of Italian origin; therefore, it is possible that interbreeding resulted in a higher hereditary predisposition to rheumatic diseases. Specific studies of the Chaima population, including genetic factors, are needed in order to establish causal links.

The most frequent comorbidity in all three ethnic groups was dental caries (44.0 %) followed by parasitic diseases (16.9 %). In addition, a high consumption of alcohol (13.9 %) and tobacco (13 %) was recorded. There were lower prevalences of hypertension and diabetes mellitus than in the indigenous population of Guatemala [37]. However, the Chaima (16.6 %) and Kari’ña (17.5 %) groups showed a higher prevalence of hypertension than in the Warao group (1.2 %), probably related to their more Westernized lifestyles. The Warao ethnic group has preserved their traditions and customs better.

Patients with osteoarthritis were predominantly female (62.6 %) and mean age was 62.4 years, compared with 84.1 % and 64 years, respectively, in Venezuelan non-indigenous patients [40]. Low back pain was more common in young men, and as in the group with osteoarthritis, showed high adaptation to pain and little limitation on their everyday lives. Rheumatoid arthritis and undifferentiated arthritis predominated in women (87.5 %; mean age, 83.3 years), below that reported in Chilean patients (Mapuches) [38].

This is the first study conducted in Venezuelan indigenous populations of the prevalence of MSK disorders and rheumatic diseases, and the methodology applied allowed us to obtain reliable results, which will be submitted to the pertinent health authorities. The results give some indication of the public health needs of the communities, and any policies should consider the epidemiological, cultural, and language differences of each ethnic group.

Venezuela was declared an illiteracy-free country by Unesco in 2003 [41], its population has free access to health and education, and the overall schooling rate stands at 93.6 %, but only 69.5 % of the indigenous population of Monagas state is literate. There are few institutions for formal education within the indigenous geographical area (seven primary education centers), and a mean schooling of 4.5 years was found in the present study, resulting in little professional or technical expertise in the indigenous population (Table 1). There were also limitations in the approach to health issues, determined by cultural aspects, difficult health care access for some communities, and insufficient hospital facilities in the communities.

This study had some limitations, such as operational and logistical constraints, because some communities were very remote in quite inaccessible places. There was over-representation of women, especially in Kari’ña communities. The reasons for this selection bias related to the following. (1) There are greater numbers of women in homes in indigenous areas, because men in Chaima and Kari’ña communities work in the fields and many Warao men fish. (2) On Saturday, men often left to buy food or sell their products in markets outside their communities. (3) Field work was not possible on Sundays because nearly everyone went to church. Despite the strategies to avoid bias, it was not possible to avoid the above representation. Language was also a problem in the case of the Warao and required the participation of bilingual male nurses. Another limitation inherent to the cross-sectional study design was the inability to determine causality, but prevalence estimates were obtained, and this was the main objective of the study.

In conclusion, this study established that the prevalence of MSK disorders and rheumatic diseases was high in the three indigenous groups. The Chaima indigenous group reported a higher prevalence of rheumatic diseases. Osteoarthritis was most prevalent in the Kari’ña and Chaima groups, followed by low back pain, which had a high prevalence in all three groups. Rheumatoid arthritis was highest in the Chaima population. This study provides useful data for the planning of health programs adapted to take into account traditional practices of indigenous communities without violating their values or ancestral beliefs.
